# Comparison of tumour-based (Petersen Index) and inflammation-based (Glasgow Prognostic Score) scoring systems in patients undergoing curative resection for colon cancer

**DOI:** 10.1038/sj.bjc.6604926

**Published:** 2009-02-10

**Authors:** C S D Roxburgh, J E M Crozier, F Maxwell, A K Foulis, J Brown, R F McKee, J H Anderson, P G Horgan, D C McMillan

**Affiliations:** 1 University Department of Surgery, University of Glasgow – Faculty of Medicine, Royal Infirmary, Glasgow, UK; 2 Department of Pathology, Glasgow Royal Infirmary, Glasgow, UK; 3 Beatson Oncology Centre, Glasgow, UK

**Keywords:** colorectal cancer, curative resection, Petersen Index, Glasgow Prognostic Score, survival

## Abstract

After resection, it is important to identify colon cancer patients, who are at a high risk of recurrence and who may benefit from adjuvant treatment. The Petersen Index (PI), a prognostic model based on pathological criteria is validated in Dukes’ B and C disease. Similarly, the modified Glasgow Prognostic Score (mGPS) based on biochemical criteria has also been validated. This study compares both the scores in patients undergoing curative resection of colon cancer. A total of 244 patients underwent elective resection between 1997 and 2005. The PI was constructed from pathological reports; the mGPS was measured pre-operatively. The median follow-up was 67 months (minimum 36 months) during which 109 patients died; 68 of them from cancer. On multivariate analysis of age, Dukes’ stage, PI and mGPS, age (hazard ratio, HR, 1.74, *P*=0.001), Dukes’ stage (HR, 3.63, *P*<0.001), PI (HR, 2.05, *P*=0.010) and mGPS (HR, 2.34, *P*<0.001) were associated independently with cancer-specific survival. Three-year cancer-specific survival rates for Dukes’ B patients with the low-risk PI were 98, 92 and 82% for the mGPS of 0, 1 and 2, respectively (*P*<0.05). The high-risk PI population is small, in particular for Dukes’ B disease (9%). The mGPS further stratifies those patients classified as low risk by the PI. Combining both the scoring systems could identify patients who have undergone curative surgery but are at high-risk of cancer-related death, therefore guiding management and trial stratification.

Colorectal cancer is the second most common cause of cancer death in Western Europe and North America. Each year in the United Kingdom, there are ∼35 000 new cases and 16 000 deaths attributable to this disease. Colon cancer accounts for the majority of disease with ∼22 000 new cases and over 10 000 deaths per year (Cancerstats, 2004). Overall survival is poor; even in those patients who undergo resection with curative intent, only half survive 5 years (McArdle and Hole, 2002).

Although Dukes’ stage is widely used to predict outcome in colon cancer, it is also recognised that the survival of patients within the staging categories is variable, particularly those with Dukes’ B or T3/4 N0 tumours. There is a particular interest in identifying subgroups of patients, with either Dukes’ stage B or stage C disease with only one positive node, who may be at a relatively high or low risk, respectively, of developing recurrent cancer and therefore may or may not benefit from adjuvant chemotherapy (Morris *et al*, 2007).

Consequently, considerable effort has been directed at refining prognostic criteria. For example, numerous molecular-based factors have been evaluated (Graziano and Cascinu, 2003). Clinically useful factors should be routinely available, well standardised and validated in a variety of different patient cohorts. However, few molecular-based factors satisfy these criteria and have been incorporated into routine clinical practice. There remains a continuing need to identify clinically relevant factors that would improve the prediction of survival in patients undergoing potentially curative surgery for colon cancer.

A score based on four routinely reported pathological criteria (vascular invasion, peritoneal involvement, margin involvement and tumour perforation) and the PI has been reported to predict cancer-specific outcome in Dukes’ B colon cancer (Petersen *et al*, 2002). More recently, the PI has been validated as a prognostic score in patients undergoing potentially curative resection for both Dukes’ B and C cancer of the colon and the rectum (Morris *et al*, 2007). Similarly, an inflammation-based score, based on two routinely measured acute phase proteins (C-reactive protein and albumin) and the Glasgow Prognostic Score (GPS) has been reported to predict the cancer-specific outcome in Dukes’ B colon cancer (McMillan *et al*, 2007). The GPS has recently been validated as a prognostic score in patients undergoing potentially curative resection for both Dukes’ B and C cancer of the colon (Ishizuka *et al*, 2007). To date, the relationship between the PI and GPS has not been examined. Moreover, the application of both scores to a single cohort of colon cancer patients has not earlier been undertaken.

The aim of this study was to compare the prognostic value of the tumour (PI) and inflammation-based (GPS) scoring systems in patients undergoing resection for colon cancer.

## Materials and methods

Patients with histologically proven colon cancer who, on the basis of laparotomy findings and pre-operative abdominal computed tomography, were considered to have undergone a potentially curative resection between January 1997 and July 2005 in a single surgical unit at the Royal Infirmary, Glasgow were included in this study. These were consecutive, elective patients, who were entered prospectively into a maintained database. The exclusion criteria were: (i) emergency surgery, (ii) death within 30 days of surgery, (iii) clinical evidence of infection or other inflammatory conditions, such as inflammatory bowel disease or rheumatoid arthritis. The tumours were staged using the conventional Dukes’ classification (Dukes and Bussey, 1958).

The Petersen Index (PI) was constructed from the scores allocated to the four selected pathological variables present in a tumour specimen. Intra or extramural vascular invasion, peritoneal involvement and margin involvement were allocated a score of 1, and tumour perforation was allocated a score of 2. The cumulative total is calculated and the PI considered low risk, in which the score is between 0 and 1 and high risk with the score between 2 and 5 (Petersen *et al*, 2002; Morris *et al*, 2007).

Blood samples were taken for routine laboratory measurements of albumin and C-reactive protein measurement before surgery. This is the standard practice in all cancer patients in our institution. The coefficient of variation for these methods, over the range of measurement, was <5% as established by routine quality control procedures. The GPS was constructed as earlier described (Forrest *et al*, 2003). Briefly, patients with both an elevated C-reactive protein (>10 mg l^−1^) and hypoalbuminaemia (<35 g l^−1^) were allocated a score of 2. Patients in whom only one of these biochemical abnormalities was present were allocated a score of 1. Moreover, patients in whom neither of these abnormalities was present were allocated a score of 0. Recently, however, the GPS has been modified based on evidence that hypoalbuminaemia, in patients with colorectal cancer without an elevated C-reactive protein concentration, had no significant association with cancer-specific survival. Therefore, patients with an elevated C-reactive protein were assigned a modified GPS (mGPS) of 1 or 2 depending on the absence or presence of hypoalbuminaemia (McMillan *et al*, 2007).

The provision of adjuvant treatment was at the discretion of the oncologist managing the patient after the multi-disciplinary team assessment. Therefore, all biochemical and pathological results, as well as patient co-morbidities were available to the oncologist for making such decisions regarding adjuvant treatment.

This study was approved by the Research Ethics Committee, Royal Infirmary, Glasgow.

### Statistics

The grouping of variables was carried out using standard thresholds. Univariate survival analysis was carried out using the Kaplan–Meier method with the log-rank test. Multivariate survival analysis and calculation of hazard ratios (HRs) were carried out using Cox's proportional-hazards model. A stepwise backward procedure was used to derive a final model of the variables that had a significant independent relationship with survival. To remove a variable from the model, the corresponding *P*-value had to be >0.05. Deaths up to 1 August 2008 were included in the analysis. Analysis was carried out using the SPSS software (SPSS Inc., Chicago, IL, USA).

## Results

Baseline clinico-pathological characteristics and the relationship with a 5-year survival rate of the patients (*n*=244), who underwent curative surgery for colon cancer, are shown in Table 1. The majority of patients were 65 years or older (73%), were male (52%) and had Dukes’ stage A or stage B disease (59%). Fifty-six (23%) patients received adjuvant chemotherapy. The median number of lymph nodes sampled was 14 (range 3–52) for Dukes’ B tumours and 14 (range 3–34) for Dukes’ C tumours. A majority of the patients had no evidence of vascular invasion (67%), peritoneal involvement (74%), resection margin involvement (91%) and tumour perforation (97%), and had a low-risk PI (87%). Moreover, a majority of the patients had C-reactive protein (51%) and albumin (83%) concentrations in the normal range and a normal mGPS (51%). Of the 40 patients with hypoalbuminaemia, 31 (78%) had an elevated C-reactive protein concentration.

The minimum follow-up was 36 months; the median follow-up of the survivors was 67 months. No patients were lost to follow-up. During this period, 68 patients died of their cancer and a further 41 patients died of intercurrent disease. The univariate survival analysis for baseline clinico-pathological characteristics is shown in Table 1. On the univariate survival analysis of individual variables, age (*P*<0.001), Dukes’ stage (*P*<0.001), vascular invasion (*P*<0.001), peritoneal involvement (*P*<0.001), resection margin involvement (*P*<0.001), tumour perforation (*P*<0.005), C-reactive protein (*P*<0.001) and albumin (*P*<0.05) were associated significantly with overall survival. Both the PI (*P*<0.001) and the mGPS (*P*<0.001) were associated significantly with overall survival (Table 1).

Furthermore, on the univariate survival analysis of individual variables, age (*P*<0.001), Dukes’ stage (*P*<0.001), vascular invasion (*P*<0.001), peritoneal involvement (*P*<0.001), resection margin involvement (*P*<0.001), tumour perforation (*P*<0.005), C-reactive protein (*P*<0.001) and albumin (*P*<0.005) were associated significantly with cancer-specific survival. On multivariate analysis of these significant variables, age (HR, 1.80, 95% CI, 1.30–2.49, *P*<0.001), Dukes’ stage (HR, 3.14, 95% CI, 1.82–5.40, *P*<0.001), vascular invasion (HR, 2.18, 95% CI, 1.25–3.82, *P*=0.006), C-reactive protein (HR, 2.09, 95% CI, 1.20–3.65, *P*=0.010) and albumin (HR, 2.33, 95% CI, 1.30–4.17, *P*=0.004) were associated independently with cancer-specific survival. On the multivariate analysis of age, Dukes’ stage, PI and mGPS, age (HR, 1.74, 95% CI, 1.27–2.39, *P*=0.001), Dukes’ stage (HR, 3.63, 95% CI, 2.13–6.18, *P*<0.001), PI (HR, 2.05, 95% CI, 1.19–3.56, *P*=0.010) and mGPS (HR, 2.34, 95% CI, 1.65–3.31, *P*<0.001) were associated independently with cancer-specific survival.

The multivariate survival analysis in patients with Dukes’ stage B and stage C disease is shown in Table 2. In those patients with Dukes’ B stage disease, age (*P*<0.05), PI (*P*<0.001) and mGPS (*P*<0.01) were associated independently with cancer-specific survival. In those patients with Dukes’ C stage disease, age (*P*<0.05) and mGPS (*P*<0.001) were associated independently with cancer-specific survival.

**Figure 1:**
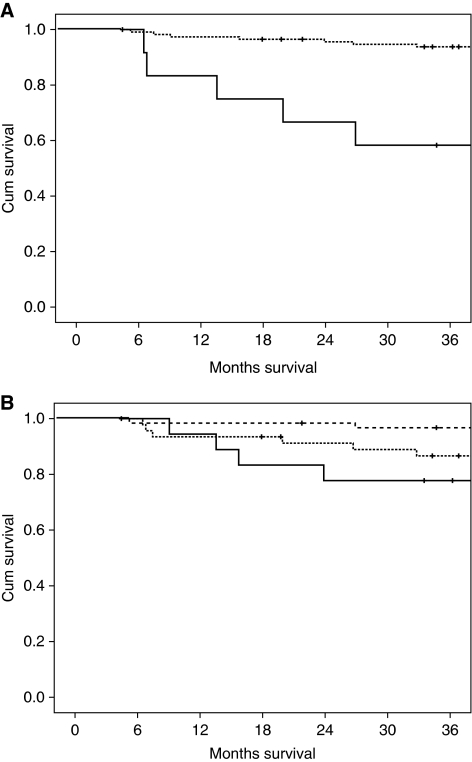
(**A**) The relationship between low- and high-risk Petersen Index (from top to bottom) and cancer-specific survival in Dukes’ B colon cancer patients (*P*<0.001). (**B**) The relationship between increasing modified Glasgow Prognostic Score (mGPS) (from the top to bottom) and cancer-specific survival in Dukes’ B colon cancer patients (*P*<0.05).

**Figure 2:**
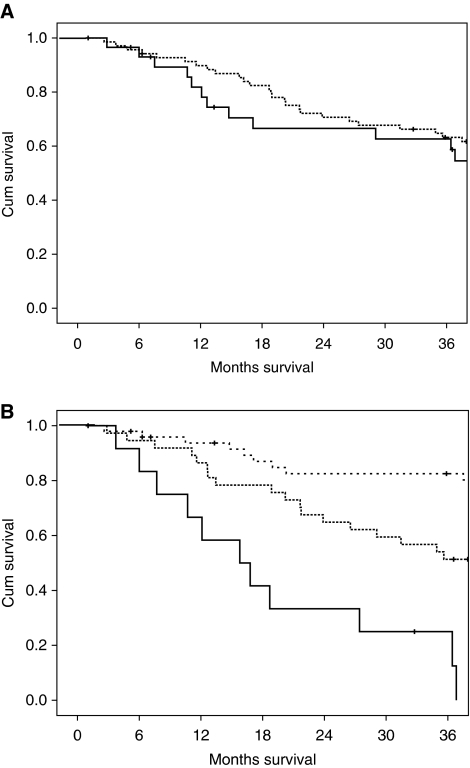
(**A**) The relationship between low- and high-risk Petersen Index (from top to bottom) and cancer-specific survival in Dukes’ C colon cancer patients (*P*=0.195). (**B**) The relationship between increasing modified Glasgow Prognostic Score (mGPS) (from the top to bottom) and cancer-specific survival in Dukes’ C colon cancer patients (*P*<0.001).

The relationships between the PI and mGPS and cancer-specific survival in Dukes’ B and C colon cancer are shown in Figures 1A and B and Figures 2A and B, respectively. The 3-year cancer-specific survival rate for patients with the low-risk PI and Dukes’ B stage disease was 98, 92 and 82% for mGPS of 0, 1 and 2, respectively (*P*<0.05; Table 3). The 3-year cancer-specific survival rate in all patients with Dukes’ C stage disease and a low-risk PI was 84, 46 and 10% for a GPS of 0, 1 and 2, respectively (*P*<0.001).

## Discussion

The PI was reported initially in Dukes’ B colon cancers (Petersen *et al*, 2002). To date only one other study has validated the PI as a prognostic score in Dukes’ B and C colon cancer, as well as rectal cancer (Morris *et al*, 2007). The results of this study further validate the PI in a different population of patients undergoing potentially curative resection for colon cancer. Of the 244 colon cancer patients included in this study, only 17% were classified as having a high-risk PI. The present PI high-risk population among Dukes’ B cases was 9%; smaller than the 29% of colon cancer cases originally reported by Petersen (Petersen *et al*, 2002), but is more comparable with the recent study by Morris *et al* (Morris *et al*, 2007), who also reported 9% of Dukes’ B colon cancers and rectal cancers as having a high-risk PI.

The basis of these differences in the classification of high-risk PI between the studies is unclear. However, it may reflect differences in case mix or variability in reporting those factors that form the PI and discriminate between Dukes’ B and C cases. In the Petersen study of Dukes’ B colon cancers, the prevalence of venous invasion (extra and intramural) was 30%; in Morris’ paper on Dukes’ B and C patients with both colon and rectal cancers, the prevalence of extramural venous was14% and in this paper on colon cancers, venous invasion was seen in 33% of colonic resections. In the three studies, peritoneal involvement was seen in 42 (Petersen), 14 (Morris) and 26%, respectively (this study). The number of lymph nodes can affect the Dukes’ staging and the mean number of lymph nodes harvested was 21, 11 and 14 in the three studies. Finally, a recent study from Australia reports a review of the slides by a single expert pathologist of 82 patients reported to have Dukes’ B cancer, but with no evidence of either venous invasion or serosal involvement. Serosal involvement and/or venous invasion were identified on review in 32% and these findings correlated with survival (Stewart *et al*, 2007).

In spite of these drawbacks in pathology reports, both our study and that of Morris and co-workers highlight the prognostic value of the PI. In particular, given that the results of both the studies were drawn from cases dissected and reported by a number of pathologists, including trainee pathologists, they are likely to be representative of ‘real world’ pathology reporting used to inform multi-disciplinary team meetings of high-risk patients with colon cancer. Therefore, we would recommend the PI should be reported routinely in patients having undergone resection for Dukes’ B colon cancer, for whom it was designed, in which the HR for survival in our study was ∼10.

Our study shows for the first time that both tumour- (PI) and inflammation-based (GPS) scoring systems have independent prognostic value in patients undergoing potentially curative resection for colon cancer. Although the PI measurement is subject to variation in reporting, the pre-operative mGPS, based on standard reliable laboratory measurements is objective and, therefore, there is likely to be little variation in reporting.

It is of interest to consider how these results might be combined in a clinical context. At present, patients with Dukes’ C tumours are offered adjuvant chemotherapy, whereas those patients with Dukes’ A tumours are not. All the relevant studies concur that the PI identifies Dukes’ B patients who are at high risk, and arguably these patients should also be offered chemotherapy. Morris has shown that patients with single node positive Dukes’ C tumours had a better prognosis than patients with Dukes’ B tumours with a PI 1. In this study, among patients with Dukes’ B tumours and a low-risk PI, a high-risk mGPS indicated a statistically significant poorer prognosis when compared with patients with pathologically similar tumours who had a low-risk mGPS (Table 3). Such high-risk patients may therefore be thought to benefit from adjuvant chemotherapy.

The utility of the PI in predicting the response to chemotherapy is not, to our knowledge, known. In contrast, there is evidence that an elevated C-reactive protein of the mGPS not only identifies those patients who are at an increased risk of recurrent disease, but also those patients who are likely to benefit from adjuvant chemotherapy (Crozier *et al*, 2006). Therefore, on the basis of the available evidence, the mGPS should be included, together with the PI, in the post-operative, multi-disciplinary assessment of patients with primary operable colon cancer and the stratification of patients entering randomised trials of adjuvant chemotherapy.

The basis of the independent relationship between an elevated mGPS before surgery and poor long-term cancer-specific survival in patients with primary operable colon cancer is not clear. A plausible explanation is that an elevated mGPS may reflect compromised cell-mediated immunity, as an elevated C-reactive protein and hypoalbuminaemia are associated with lymphocytopenia (Nozoe *et al*, 2000) and with an impaired T-lymphocytic response in the tumour (Canna *et al*, 2005). Furthermore, the presence of an elevated C-reactive protein concentration and hypoalbuminaemia have also been shown to be associated with the upregulation of components of innate immune system, including complement and macrophage functions (Coussens and Werb, 2002; Du Klos and Mold, 2004). In addition, it is known that as part of the systemic inflammatory response, there is a release of pro-inflammatory cytokines and growth factors which may promote tumour growth (Abramovitch *et al*, 1999; Canna *et al*, 2007). Therefore, the mGPS may reflect host responses that impact prognosis in colon cancer, whereas the PI might be considered to provide prognostic information on tumour behaviour.

In summary, the results of this study validate the use of the PI in predicting cancer-specific survival in patients undergoing elective potentially curative for Dukes’ B colon cancer. Furthermore, the results indicate that the mGPS further stratifies those patients with Dukes’ B and single node positive Dukes’ C cancers, classified as low risk by the PI. The PI and mGPS scoring systems could, therefore, be combined at a multi-disciplinary meeting to identify those patients with colorectal cancer who have undergone potentially curative surgery but are at high risk of cancer related death.

## Figures and Tables

**Table 1:** Clinico-pathological characteristics in patients undergoing potentially curative resection for colon cancer: univariate survival analysis

	**Patients 244 (%)**	**Overall 3-year survival rate % (s.e.)**	***P*-value (log-rank)**	**Cancer 3-year survival rate % (s.e.)**	***P*-value (log-rank)**
*Age*					
<65 years	65 (27)	95 (3)		95 (3)	
65–74 years	72 (29)	76 (5)		84 (4)	
>75 years	107 (44)	61 (5)	<0.001	68 (5)	<0.001
					
*Sex*					
Female	118 (48)	70 (4)		79 (4)	
Male	126 (52)	79 (4)	0.416	81 (4)	0.832
					
*Dukes’ stage*					
A	18 (7)	94 (5)		100 (0)	
B	127 (52)	85 (3)		90 (3)	
C	99 (41)	58 (5)	0.001	63 (5)	<0.001
					
*Adjuvant therapy*					
No	88 (77)	74 (3)		81 (3)	
Yes	56 (23)	77 (6)	0.028	77 (6)	0.906
					
*Date of surgery*					
1997–2001	116 (48)	77 (4)		81 (4)	
2002–2005	128 (52)	73 (4)	0.514	79 (4)	0.773
					
					
*Pathological characteristics*					
* Vascular invasion*					
No	163 (67)	83 (3)		86 (3)	
Yes	81 (33)	58 (5)	<0.001	67 (5)	<0.001
					
* Peritoneal involvement*					
No	180 (74)	81 (3)		86 (3)	
Yes	64 (26)	51 (6)	0.001	64 (6)	<0.001
					
* Margin involvement*					
No	221 (91)	76 (3)		82 (3)	
Yes	23 (9)	57 (10)	<0.001	60 (10)	<0.001
					
* Tumour perforation*					
No	308 (98)	75 (3)		81 (3)	
Yes	6 (2)	50 (25)	0.001	50 (25)	0.002
					
*Biochemical characteristics*					
* C-reactive protein*					
⩽10 mg l^−1^	125 (51)	86 (4)		92 (3)	
>10 mg l^−1^	119 (49)	63 (4)	<0.001	68 (4)	<0.001
					
* Albumin*					
⩾35 g l^−1^	204 (83)	78 (3)		83 (3)	
<35 g l^−1^	40 (17)	58 (8)	0.001	67 (8)	0.004
					
*Petersen Index*					
Low risk	203 (87)	79 (3)		84 (3)	
High risk	41 (13)	54 (8)	<0.001	61 (8)	<0.001
					
					
*mGPS*					
Low risk (0)	125 (51)	86 (3)		92 (3)	
Intermediate (1)	88 (36)	68 (5)		72 (5)	
High risk (2)	31 (13)	48 (9)	<0.001	57 (9)	<0.001

mGPS=modified Glasgow Prognostic Score; s.e.=standard error of mean.

**Table 2:** Clinico-pathological characteristics and 3-year cancer specific survival in patients undergoing potentially curative resection for Dukes’ B and Dukes’ C colon cancer: multivariate survival analysis

**Dukes’ B**	***n*=127 (%)**	**Three-year survival % (s.e.)**	**Hazard ratio (95% CI)**	***P*-value**
*Age*				
<65 years	37 (29)	100 (0)		
65–74 years	40 (31)	92 (4)		0.034
>75 years	50 (40)	81 (6)	1.87 (1.05–3.34)	
				
*Sex*				
Female	61 (48)	90 (4)		
Male	66 (62)	91 (4)	0.99 (0.39–2.51)	0.984
				
*Adjuvant therapy*				
No	111 (87)	92 (3)		
Yes	16 (13)	81 (10)	0.98 (0.22–4.44)	0.979
				
*Petersen Index*				
Low	115 (91)	94 (2)		
High risk	12 (9)	58 (14)	9.61 (3.27–28.26)	<0.001
				
*mGPS*				
Low 0	62 (49)	97 (2)		
Intermediate 1	47 (37)	87 (5)		
High risk 2	18 (14)	78 (10)	2.15 (1.19–3.87)	0.010
				

**Dukes’ C**	***n*=99 (%)**	**Three-year survival % (s.e.)**	**Hazard ratio (95% CI)**	***P*-value**
*Age*				
<65 years	25 (25)	88 (6)		
65–74 years	25 (25)	66 (10)		
>75 years	49 (50)	48 (7)	1.72 (1.08–2.75)	0.022
				
*Sex*				
Female	49 (49)	62 (7)		
Male	50 (51)	64 (7)	1.25 (0.68–2.31)	0.477
				
*Adjuvant therapy*				
No	59 (60)	54 (7)		
Yes	40 (40)	75 (7)	0.92 (0.42–2.00)	0.832
				
*Petersen Index*				
Low	70 (71)	63 (6)		
High risk	29 (29)	63 (9)	1.16 (0.60–2.24)	0.655
				
*mGPS*				
Low 0	50 (51)	83 (6)		
Intermediate 1	37 (32)	51 (8)		
High risk 2	12 (17)	24 (13)	2.80 (1.80–4.44)	<0.001

CI=confidence interval; mGPS=modified Glasgow Prognostic Score; s.e.=standard error of mean.

**Table 3:** The relationship between the low-risk Petersen Index, and the mGPS with a 3-year survival (%) in patients undergoing potentially curative resection for Dukes’ B, single node positive Dukes’ C and Dukes’ C colon cancer

	**Petersen Index**	**Petersen Index**
**Dukes’ B**	**Low risk (*n*=115)**	**High risk (*n*=12)**
*mGPS*		
Low risk (0)	98% (*n*=56)	82% (*n*=6)
Intermediate (1)	92% (*n*=42)	40% (*n*=5)^*^
High risk (2)	82% (*n*=17)^**^	0% (*n*=1)^**^
mGPS(0–2)	94% (*n*=115)	58% (*n*=12)
		

	**Petersen Index**	**Petersen Index**
**Dukes’ C**	**Low risk (*n*=70)**	**High risk (*n*=29)**
*mGPS*		
Low risk (0)	84% (*n*=39)	76% (*n*=11)
Intermediate (1)	46% (*n*=24)^***^	62% (*n*=13)
High risk (2)	10% (*n*=7)^****^	40% (*n*=5)^***^
mGPS(0–2)	63% (*n*=70)	63% (*n*=29)

mGPS=modified Glasgow Prognostic Score.

^*^P<0.1, ^**^P<0.05, ^***^P<0.01, ^****^P<0.001: Association between the increasing mGPS and cancer-specific survival on univariate analysis.
